# Role of biomarkers as predictors of acute kidney injury and mortality in decompensated cirrhosis

**DOI:** 10.1038/s41598-019-51053-8

**Published:** 2019-10-10

**Authors:** Sang Kyung Jo, Jihyun Yang, Sang Muk Hwang, Myung Seok Lee, Sang Hoon Park

**Affiliations:** 1 Division of Nephrology, Department of Internal Medicine, Korea University Medical College, Seoul, Korea; 2 Division of Gastroenterology and Hepatology, Department of Internal Medicine, Kangnam Sacred Heart Hospital, Hallym University College of Medicine, Seoul, Korea

**Keywords:** Predictive markers, Liver cirrhosis

## Abstract

Evidence suggests that novel biomarkers predict acute kidney injury (AKI) development and outcome earlier than serum creatinine. The aim of this study was to determine the incidence and prognosis of AKI in decompensated cirrhotic patients, and also assess the usefulness of plasma cystatin C, urine neutrophil gelatinase associated lipocalin (NGAL), tissue inhibitor of metalloproteinase-2 (TIMP-2) and insulin-like growth factor-binding protein 7 (IGFBP7) in early prediction of AKI and mortality. Single-center, prospective observational study enrolling decompensated cirrhotic patients without AKI at the time of admission. Of 111 patients with decompensated cirrhosis, 45 (40.5%) developed AKI while hospitalized. Even with 53.3% being transient (stage 1), mortality was significantly higher in AKI than non-AKI patients (46.5% vs. 25%, p = 0.02). Plasma cystatin C and urine NGAL, but not urine [TIMP-2]·[IGFBP7] at the time of admission were found to be independent early predictors of AKI. Substitution of cystatin C for creatinine significantly improved the model for end-stage liver disease (MELD) score accuracy for mortality prediction. The incidence of AKI is high and is associated with high mortality in decompensated cirrhotic patients. Plasma cystatin C and urine NGAL are useful for early detection of AKI. MELD-cystatin C, rather than original MELD, improves predictive accuracy of mortality.

## Introduction

Acute kidney injury (AKI) is a clinical syndrome characterized by a rapid decline in kidney function due to hemodynamic alteration or structural damage. As it frequently complicates the course of many chronic diseases with worse outcome, early detection of this devastating complication is critical^1–5^. However, since AKI diagnosis is still dependent on increase in serum creatinine levels or decreased urine output, both of which lack sensitivity, research over the last several decades has aimed to discover and validate novel biomarkers for earlier AKI detection^6,7^. AKI is also known to be a frequent complication in advanced liver diseases, occurring in 16.8–50% of admitted cirrhotic patients^1,5,8,9^. However, increased volume of distribution, decreased creatinine production from muscle wasting, and liver dysfunction frequently found in these patients further compromise diagnostic and prognostic value of serum creatinine.

Therefore, in this study, we tested the usefulness of plasma cystatin C, urine neutrophil gelatinase associated lipocalin (NGAL), urine tissue inhibitor of metalloproteinase-2 (TIMP-2), and insulin-like growth factor-binding protein 7 (IGFBP7) as early independent predictors of AKI development and outcome in decompensated cirrhotic patients. Cystatin C is a low molecular weight cysteine protease inhibitor, produced by all nucleated cells and freely filtered by glomeruli^10^. As cystatin C is less affected by age, gender, or muscle mass, it has already been shown to be a better marker of glomerular filtration rate (GFR). NGAL is a 25 kDa protein generally expressed in neutrophils and its expression markedly increases following injury to kidney tubular cells^11^. Although many studies have shown NGAL to be a novel biomarker of early and sensitive AKI detection, its potential usefulness in decompensated cirrhosis remains unclear^12–16^. We also investigated the possible value of urinary [TIMP-2]·[IGFBP7], a recently discovered biomarker, in predicting AKI and mortality. Nephrocheck®, which simultaneously detects TIMP-2 and IGFBP7, has been approved by the United States Food and Drug Administration as a potential aid in the prediction of moderate to severe AKI within 12 hours in critically ill patients^17–19^. In addition, the accuracy of cystatin C-based MELD, compared to creatinine-based MELD, in predicting mortality was also determined.

## Results

### Patient characteristics

**Figure 1:**
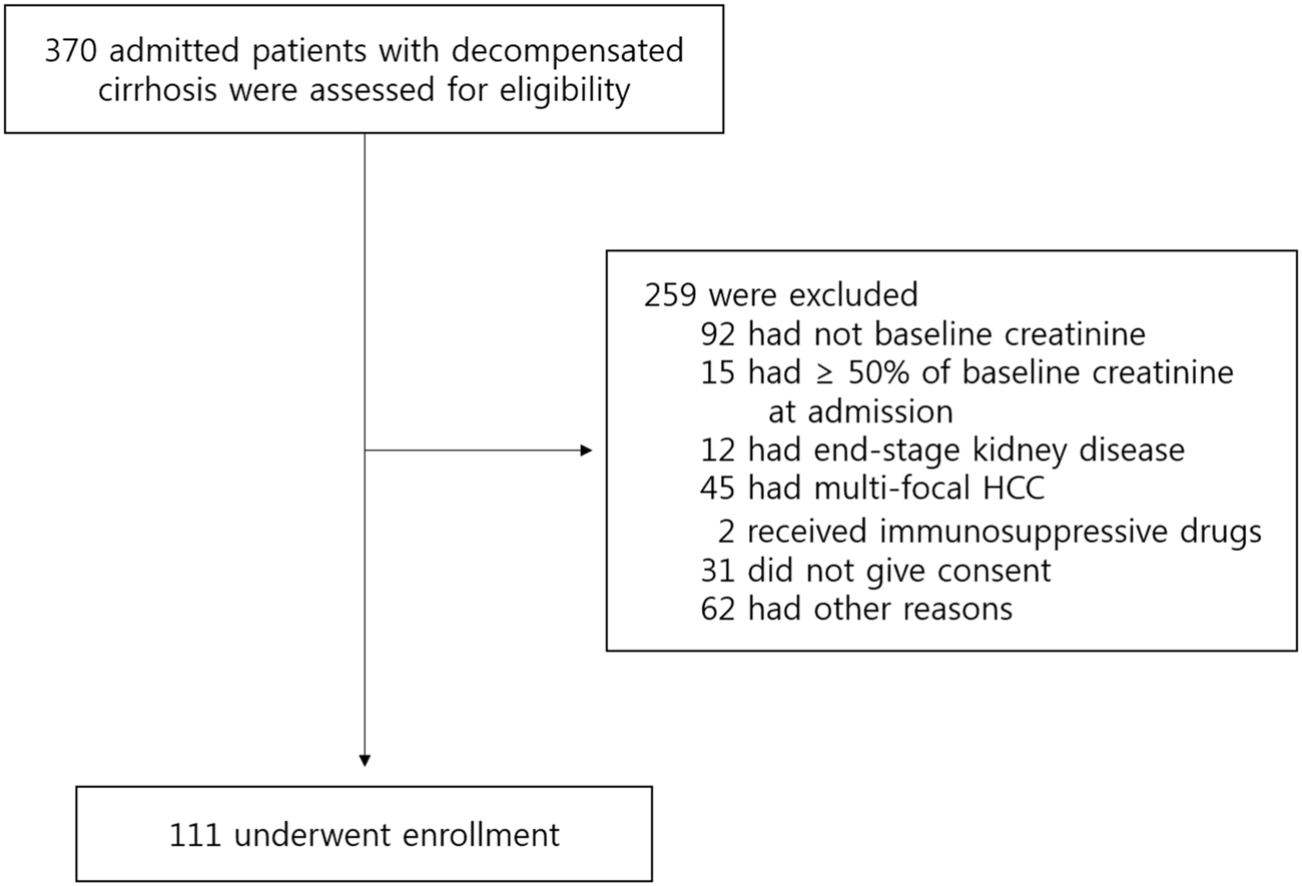
Flow chart of the study population. Among a total of 370 admitted patients with decompensated cirrhosis who were initially assessed for the inclusion, 259 patients were excluded due to unknown or abnormal baseline renal function, development of AKI at the time of admission, taking immunosuppressive drugs from previous transplantation or refusal to give consent etc. and 111 patients were finally enrolled.

Among a total of 370 patients who were initially assessed for the inclusion, the 111 acutely decompensated cirrhotic patients with known baseline renal function but without AKI at the time of admission were prospectively recruited (Fig. 1). The mean age was 58.9 ± 12.2 years, and 71.2% were men. The causes of decompensation included variceal bleeding, ascites, hepatic encephalopathy, spontaneous bacterial peritonitis, and other infections (Table 1). The underlying etiologies of cirrhosis were alcohol (n = 51), hepatitis B virus (n = 38), hepatitis C virus (n = 17) or combined viral and alcohol or cryptogenic (n = 5).

**Table 1:** Baseline characteristics of no-AKI *vs.* AKI.

	Total (111)	No-AKI (66)	AKI (45)	*p-value*
Age	58 [29–85]	59 [29–85]	56 [34–84]	0.95
Sex (male)	79 (71.2%)	49 (74.2%)	30 (66.7%)	0.256
Hypertension	24 (21.6%)	15 (22.7%)	9 (20%)	0.461
Diabetes mellitus	34 (30.6%)	23 (34.8%)	11 (24.4%)	0.169
Hepatic encephalopathy	21 (18.9%)	14 (21.2%)	7 (15.6%)	0.312
Ascites	76 (68.5%)	42 (63.6%)	34 (75.6^)	0.131
Esophageal varix	93 (83.7%)	52 (78.8%)	41 (95.3%)	0.014
Spironolactone use	40 (36%)	22 (33%)	18 (40%)	0.3
Furosemide use	36 (32.4%)	20 (30.3%)	16 (35.6%)	0.35
Dual diuretics (S + F)	35 (31.5%)	19 (28.8%)	16 (35.6%)	0.29
Systolic BP (mmHg)	120 [55–175]	120 [93–175]	113 [55–147]	0.002
Diastolic BP (mmHg)	71 [31–126]	73 [47–126]	70 [31–104]	0.08
Charlson comorbidity index	8 [5–14]	5 [1–14]	6 [1–11]	0.005
MELD score	12.73 ± 7.5	10.9 ± 6.5	15.3 ± 8.6	0.002
Treatment				
Albumin	27 (24.3%)	11 (16.7%)	16 (35.6%)	0.026
Terlipressin	20 (18%)	8 (12.1%)	12 (26.7%)	0.045
Fresh frozen plasma	30 (27%)	13 (19.7%)	17 (37.8%)	0.035
Packed red blood cells	22 (19.8%)	9 (13.6%)	13 (28.9%)	0.048
Platelet concentrates	4 (3.6%)	1 (1.5%)	3 (6.7%)	0.153
Hemodialysis	1 (0.9%)	0	1 (2.2%)	−
Mortality	36 (32.4%)	16 (25.4%)	20 (46.5%)	0.02
BUN (mg/dL, admission)	21.3 [2.3–101.5]	14.6 ± 8.9	16.8 ± 14.3	0.35
Creatinine (mg/dL, admission)	79 ± 0.5	0.78 ± 0.33	0.8 ± 0.5	0.7
BUN (mg/dL, peak)	24.8 ± 21.4	18.1 ± 11.6	25.8 ± 20.6	0.01
Creatinine (mg/dL, peak)	1.1 ± 0.8	0.82 ± 0.3	1.17 ± 0.7	0.001
Serum Na (mEq/L)	132 ± 1.3	134 ± 5.6	132 ± 7.8	0.89
CRP (mg/dL)	6.3 [0–156.2]	3.9 [0–153.7]	13.9 [0–156.2]	0.04
FENa (%)	0.20 [0.2–1.96]	0.22 [0.002–1.96]	0.13 [0.006–1.56]	0.83
Urine protein/creatinine ratio	0.46 ± 1.7	0.25 ± 4.03	0.78 ± 17.1	0.107
plasma Cystatin C (mg/dL)	1.2 ± 0.6	0.99 ± 0.25	1.18 ± 1.6	0.01
Corrected NGAL (μg/gCr)	209.8 ± 465.5	56.85 ± 122.6	261.62 ± 507	0.007
[TIMP-2][IGFBP7]/1000	0.18 ± 0.8	0.1 ± 0.48	0.31 ± 1.09	0.019

(S + F): spironolactone + furosemide, BUN: blood urea nitrogen, CRP: C-reactive protein, FENa: fractional excretion of Na, NGAL: neutrophil gelatinase associated lipocalin, TIMP-2: tissue inhibitor of metalloproteinase-2, IGFBP7: insulin-like growth factor-binding protein7.

### Development of AKI

Of the 111 patients, 40.5% developed AKI. The median time from admission to peak creatinine level in AKI was 2 days (mean time; 4.72 ± 1.3 days). The AKI patient group had a higher prevalence of varices, lower systolic blood pressures, higher C-reactive protein (CRP) levels, and higher CCI (Charlson comorbidity index) and MELD scores. More than 50% of AKI episodes were stage 1 AKI (53.3%) with only modest increase in serum creatinine, and 13.3% and 33.3% of the AKI episodes were stage 2 AKI and stage 3 AKI, respectively with only one patient needing temporary dialysis (Table 1). Dehydration, bleeding and infection comprised the major etiologies of AKI (34, 26 and 24.4%, respectively) with only 4 patients of unknown causes.

Initial BUN, creatinine level, fractional excretion of sodium, urine protein/creatinine ratio, and diuretic use were not statistically different between the AKI and non-AKI groups. However, plasma cystatin C, urine NGAL, and urine [TIMP-2]·[IGFBP7] levels at the time of admission were significantly increased in the AKI group compared to the non-AKI group (Cystatin C: 1.18 ± 1.6 *vs*. 0.99 ± 0.25 mg/dL, p = 0.01, NGAL 261.62 ± 507 *vs*. 56.85 ± 122.6 μg/gCr, p = 0.007, [TIMP-2]·[IGFBP7] 0.31 ± 1.09 *vs*. 0.1 ± 0.48, p = 0.019) (Table 1).

During the hospital stay, patients were treated with terlipressin, albumin, packed red blood cells or fresh frozen plasma according to patients needs and significantly higher percentage of patients with AKI received these treatments except platelet concentrates. Eighteen out of twenty patients who received terlipressin were due to variceal bleeding (Table 1).

### Performance of novel biomarkers in early prediction of AKI

**Figure 2:**
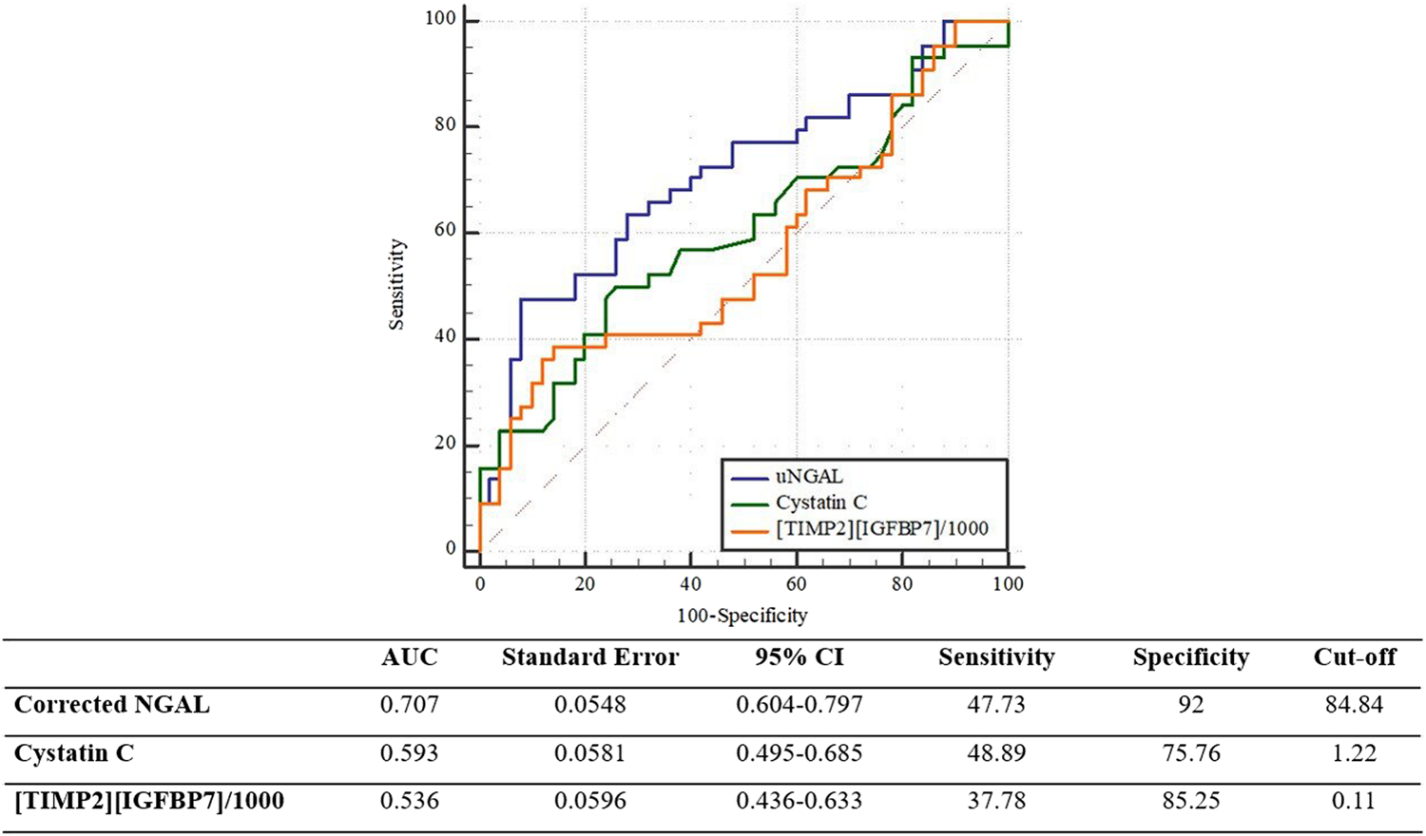
Comparison of AUCs of biomarkers for early prediction of AKI. Corrected urine NGAL is superior to plasma cystatin C or urine [TIMP2][IGFBP7]/1000 in early prediction of AKI.

To investigate the usefulness of these novel biomarkers as independent early predictors of AKI in decompensated cirrhotic patients, we performed univariate and multivariate analyses of variables associated with the development of AKI at the time of inclusion. Plasma cystatin C (OR: 2.09, 95% CI: 1.01–4.35) and urine NGAL (OR: 1.04, 95% CI: 1.01–1.05) but not [TIMP-2]·[IGFBP7] at the time of admission were found to be independent predictors of AKI in multivariate analysis after adjusting for clinical variables including age, sex, hypertension, diabetes, and diuretic use (Table 2). To further assess the predictive accuracy of these 2 biomarkers in the prediction of AKI, we calculated the area under the ROC (AUROC). The AUROC of urine NGAL showed the highest value of 0.707 (95% CI: 0.604–0.797). The cut-off value of urine NGAL in early prediction of AKI was 84.8 µg/gCr (Fig. 2).

**Table 2:** Univariate and multivariate logistic regression for predicting AKI.

	Univariate			Multivariate		
	Odds ratio	95% CI	*p*-value	Odds ratio	95% CI	*p*-value
Age	0.99	0.968–1.031	0.95			
Sex (male)	0.694	0.3–1.591	0.38			
CCI	1.06	0.91–1.23	0.42			
MELD	1.08	1.02–1.14	0.004			
CRP	1.01	1.000–1.021	0.056			
uPCR	1.28	0.85–1.93	0.23			
FENa	1.12	0.85–1.47	0.42			
Cystatin C	2.357	1.14–4.86	0.02	2.09	1.01–4.35	0.02
Corrected NGAL(μg/gCr)	1.004	1.000–1.007	0.03	1.04	1.01–1.05	0.001
[TIMP2][IGFBP7]/1000	2.335	0.433–12.607	0.32	1.135	0.551–2.34	0.73

Multivariate analysis includes demographic factors (Age, Sex, CCI) + biomarker independently.

CCI: Charlson comorbidity index, CRP: C-reactive protein, uPCR: urine protein/creatinine ratio, FENa: fractional excretion of Na, NGAL: neutrophil gelatinase associated lipocalin, TIMP-2: tissue inhibitor of metalloproteinase-2, IGFBP7: insulin-like growth factor-binding protein7.

### Mortality

Of 111 patients, 24 patients in whom the 90-day mortality could not be determined were excluded in analysis. The 90-day mortality rate was 34%. Although more than half of the AKI patients only had mild, transient stage 1 AKI, mortality was significantly higher in the AKI group than in the non-AKI group (46.5% *vs*. 25.4%, p = 0.02) with a stepwise increase accompanying increasing AKI severity (39.1% *vs*. 50.0% *vs*. 57.1%, p = 0.014) (Table 3). The patients who died within 90 days were significantly older with higher CCI, higher MELD score, and higher CRP and plasma cystatin C levels at the time of admission. However, their initial urine NGAL and [TIMP2]·[IGFBP7] values were not different (Table 3).

**Table 3:** Baseline characteristics of survivors *vs.* non-survivors.

	Total (87)	Survivors (53)	Non-survivors (34)	*p-value*
Age	59 [29–85]	56 [29–84]	64 [46–85]	0.004
Sex (male)	79 (70.5%)	35 (66%)	27 (79.4%)	0.13
Hypertension	24 (21.6%)	8 (44.4%)	10 (55.6%)	0.09
Diabetes mellitus	34 (30.6%)	16 (30.2%)	12 (35.3%)	0.39
Hepatic encephalopathy	21 (18.8%)	7 (13.2%)	9 (26.5%)	0.102
Ascites	76 (67.9%)	30 (56.6%)	28 (82.4%)	0.01
Esophageal varix	93 (83%)	44 (83%)	30(90.9%)	0.24
Spironolactone use	40 (35.7%)	17 (32.1%)	12 (35.3%)	0.47
Furosemide use	36 (32.1%)	15 (28.3%)	12 (35.3%)	0.33
Dual diuretics (S + F)	35 (31.3%)	15 (28.3%)	11(32.4%)	0.43
Systolic BP	120 [55–170]	120 [55–175]	110 [91–165]	0.19
Diastolic BP	73 [31–126]	80 [31–126]	70 [54–104]	0.07
Charlson comorbidity index	6 [1–14]	4.6 ± 1.8	7.4 ± 2.7	<0.001
MELD score	12.73[0.018–32.23]	9.6[0.018–31.09]	17.6[2.19–32.23]	<0.001
BUN (peak)	24.8 [3.9–122.8]	17 ± 11.28	26 ± 22.5	0.01
Creatinine (peak)	1.1 [0.2–5.1]	0.87 ± 0.48	1.07 ± 0.62	0.07
AKI stage				<0.001
Stage 1	19	11 (20.8%)	8 (23.5%)	
Stage 2	4	1 (1.9%)	3 (8.8%)	
Stage 3	13	1 (1.9%)	12 (35.3%)	
Serum Na	132 [113–148]	135[106–148]	133[117–142]	0.22
CRP	24.23 [0–156.2]	17.6 ± 29.6	37.6 ± 46.3	0.008
FENa	0.68 [0.003–9.64]	0.3 ± 0.38	0.34 ± 0.37	0.63
Urine protein/creatinine ratio	0.46 [0.002–13.08]	0.38 [0.003–9.5]	0.64 [0.017–2.83]	0.5
Serum Cystatin C	1.2 [0.42–4.97]	1.0 ± 0.3	1.5 ± 0.8	<0.001
Corrected NGAL (μg/gCr)	209.79 [0.69–2402.81]	135.7 [0.69–1757]	190.8 [7.16–2402]	0.23
[TIMP2][IGFBP7]/1000	0.18 [0.0002–5.27]	0.12 [0.0003–5.26]	0.32 [0.003–5.18]	0.21
MELD-cystatin C	14.3 [0.3–32.75]	12 [0.3–32.23]	20.5 [9.7–32.75]	<0.001

(S + F): spironolactone + furosemide, FENa: fractional excretion of Na, NGAL: neutrophil gelatinase associated lipocalin, TIMP-2: tissue inhibitor of metalloproteinase-2, IGFBP7: insulin-like growth factor-binding protein7.

### Performance of novel biomarkers in predicting mortality

**Figure 3:**
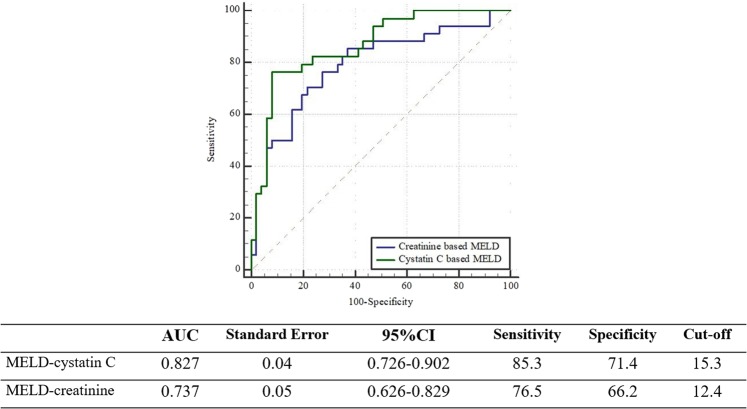
Comparison of AUCs of each scoring system for mortality prediction. Cystatin C-based MELD is more powerful in mortality prediction than creatinine-based MELD.

Older age, male sex, higher MELD score, higher CRP level, and higher plasma cystatin C were associated with 90-day mortality in the univariate analysis (Table 4). However, only the MELD score was found to be an independent predictor of mortality after adjusting multiple clinical and laboratory variables (Table 4). We then assessed the performance of MELD-cystatin C instead of creatinine in predicting mortality and found that simple substitution of cystatin C for creatinine in the MELD score significantly improved its predictive performance (Fig. 3).

**Table 4:** Univariate and multivariate cox regression for predicting mortality.

	Univariate			Multivariate		
	HR	95% CI	*p*-value	HR	95% CI	*p*-value
Age	1.05	1.014–1.091	0.006	1.058	0.966–1.063	0.58
Sex (male)	2.78	1.02–7.58	0.02	2.13	0.4–11.43	0.38
Hypertension	1.64	0.78–3.42	0.19			
Diabetes mellitus	1.48	0.7–3.1	0.3			
MELD	1.111	1.05–1.18	0.001	1.2	1.07–1.36	0.002
CRP	1.01	1.003–1.025	0.013	1.01	0.999–1.02	0.06
UPCR	1.04	0.84–1.31	0.69			
FENa	0.87	0.55–1.35	0.523			
Cystatin C	5	1.9–13.2	0.001			
Corrected NGAL	1.001	0.999–1.002	0.27			
[TIMP2][IGFBP7]/1000	1.04	0.638–1.702	0.87			
MELD-cystatin C	1.093	1.043–1.146	<0.001	1.1	1.018–1.146	0.001

Multivariate regression included Age, sex, HTN, DM, CKD, CRP and MELD or MELD using biomarker.

NGAL: neutrophil gelatinase associated lipocalin, TIMP-2: tissue inhibitor of metalloproteinase-2, IGFBP7: insulin-like growth factor-binding protein7.

## Discussion

In this prospective observational study, we demonstrated that: 1) the incidence of AKI in decompensated cirrhotic patients is high (40.5%) and mortality is higher in the AKI group than the non-AKI group, even though transient stage 1 AKI made up the majority of the AKI episodes, 2) plasma cystatin C and urinary NGAL excretion, but not urinary [TIMP2][IGFBP7], at the time of admission can independently predict the development of AKI earlier than serum creatinine, and 3) although MELD score was found to be the only independent predictor of 90-day mortality in these patients, replacement of creatinine by plasma cystatin C significantly improved the predictive performance of MELD.

AKI is a clinical syndrome of varied etiologies characterized by the acute decline of renal function and shown to be associated with poor outcome in several chronic diseases such as heart failure and liver cirrhosis. However, due to a lack of specific signs and symptoms, diagnosis of AKI still depends on changes in serum creatinine or urine output. As serum creatinine is affected by gender, age, and muscle mass, diagnostic sensitivity can be considerably inferior in cirrhotic patients due to decreased muscle mass, impaired synthesis, or dilution from expanded extracellular volume. The urine output criterion suggested by KDIGO is also substantially limited by reductions in the effective circulating volume or frequent use of diuretics in these patients. Therefore, studies focusing on the discovery and validation of novel biomarkers for earlier and more sensitive AKI detection and/or outcome prediction have been highly prioritized over the last several decades.

Although 45 of the 111 cirrhotic patients admitted due to acute decompensation developed AKI (median time from admission to peak creatinine level in AKI was 2 days), neither initial BUN, creatinine level nor fractional sodium and urea excretion at the time of admission were statistically different between the groups, thereby confirming the lack of sensitivity of these traditional parameters of renal dysfunction or damage. In contrast, plasma cystatin C and urine NGAL levels at the time of admission were significantly elevated in patients who developed AKI and were also found to be independent predictors of AKI. This is comparable to results of several other studies that demonstrated the usefulness of these biomarkers in predicting not only AKI but also the development of acute-on-chronic liver failure and mortality. In our study, the performance of urine NGAL as an early predictor of AKI was found to be superior to that of plasma cystatin C. This finding suggests that kidney tubular injury marker, rather than functional markers, may be more sensitive for early detection of AKI in advanced cirrhosis. In addition, we also tested the usefulness of urine [TIMP-2]·[IGFBP7], a recently discovered and validated novel biomarker, in our decompensated cirrhotic patient cohort. These markers represent G0–G1 phase cell cycle arrest and Nephrocheck®, an assay kit used for the assessment of the risk of developing stage 2 AKI or higher within 12 hours in critically ill patients, was approved by the United States Food and Drug Administration in 2014^18^. Although the median level of urine [TIMP-2]·[IGFBP7] was significantly higher in the AKI group than the non-AKI group, it was unable to independently predict AKI in our cohort. This is in contrast with several other research findings that showed urine [TIMP-2]·[IGFBP7] accurately predicts AKI in critically ill, platinum-treated or high-risk surgical patients and suggest that different biomarker or biomarker panels should be used in different patient groups^19^. Although the reason why the urine [TIMP-2]·[IGFBP7] fail to predict AKI is not clear in this study, production from damaged hepatocytes in chronic liver disease patients might be one possible mechanism. However, despite negative result, this is the first study testing the usefulness of urine [TIMP-2]·[IGFBP7] as an early biomarker of AKI in advanced liver disease patients.

Earlier detection of AKI by plasma cystatin C or urine NGAL in decompensated cirrhotic patients may have a huge impact on patient outcome if it leads to individually tailored therapy including more intensive monitoring of volume status and avoidance of nephrotoxic drugs such as aminoglycosides, diuretics or radio-contrast agents. Belcher *et al*. previously reported that every 12-hour delay in AKI diagnosis of hospitalized cirrhotic patients increased hospital mortality rate 2.7 times^15^. The possible usefulness of biomarker-guided interventions in AKI prevention has recently been demonstrated in the study by Meersch *et al*. in which AKI-directed care of biomarker-positive, creatinine-negative cardiac surgical patients resulted in an absolute risk reduction of 16.6% in the incidence of AKI^20^.

In our study, we confirmed that the AKI patient group had a significantly higher 90-day mortality rate than the non-AKI group despite half of AKI episodes were transient, stage 1 AKI mostly due to volume depletion or infection. The mortality rate increased in a stepwise fashion as the KDIGO stage increased (25.4%, 39.1%, 50.0%, and 57.1% for no AKI, stage1 AKI, stage 2 AKI, and stage 3 AKI, respectively) and etiology of AKI did not affect the mortality rate. This is contrary to the notion that transient mild AKI, especially prerenal azotemia mostly due to depletion of effective circulating volume has a favorable prognosis. Instead, it indicates that even KDIGO stage 1 AKI in advanced liver disease patients might be an ominous sign for poor outcome.

Although direct comparison is not possible due to a lack of dialysis-requiring AKI cases in our cohort, extremely high mortality has been reported in renal replacement therapy-requiring stage 3 AKI cirrhotic patients regardless of AKI etiology.

As expected, only higher MELD score was found to be an independent predictor of mortality in multivariate analysis. While plasma cystatin C level can predict mortality after adjusting for age, sex, comorbidity, the prothrombin time international normalized ratio (PT-INR), and bilirubin, it loses its usefulness when MELD score is included as a variable. However, we found that substituting cystatin C for creatinine significantly improves the prediction accuracy of the original MELD score that incorporates creatinine. The usefulness of plasma cystatin C in mortality prediction has been demonstrated in several studies. Markwardt *et al*. showed that initial plasma cystatin C levels predict the development of renal dysfunction, acute-on-chronic liver failure, and mortality in 429 patients who were part of the EASL-CLIF Acute-on-Chronic Liver Failure in Cirrhosis (CANONIC) study^21^. However, in a study by Finkenstedt *et al*., substitution of creatinine by cystatin C with the use of regression coefficient derived from multivariate Cox regression did not improve the predictive power of MELD^22^. Considering this controversy, the usefulness of the cystatin-based prediction model needs to be further investigated and confirmed in larger-scale studies given that cystatin C is less affected by age, sex, and muscle mass and can predict AKI earlier than creatinine.

Despite the several meaningful study findings, there are limitations to acknowledge in our study. This is a single-center study with a relatively small sample size. Furthermore, due to the recruitment of hospitalized cirrhotic patients, these results cannot be applied to all stages of cirrhosis including acute-on-chronic liver failure and stable cirrhotic patients.

In conclusion, our study showed plasma cystatin C and urine NGAL, but not urine urine [TIMP-2]·[IGFBP7] to be potentially useful biomarkers in decompensated cirrhotic patients for early sensitive detection of AKI. We also showed that substituting cystatin C for creatinine may improve the performance of MELD in predicting short-term mortality.

## Methods

### Study design & patients

This study is a single-center prospective observational study of recruited patients admitted to the Division of Gastroenterology & Hepatology of Hallym University Kangnam Sacred Heart Hospital for decompensated cirrhosis (i.e., with ascites, variceal bleeding, spontaneous bacterial peritonitis, hepatic encephalopathy etc.) from May 2015 to December 2017. All procedures followed were in accordance with the ethical standards of the responsible committee on human experimentation (institutional and national) and with the Helsinki Declaration of 1975, as revised in 2008. The Institutional Review Board of Hallym University Medical Center approved the study (IRB number: 2015-06-078) and informed consent was obtained from all eligible patients. Cirrhosis was diagnosed by either liver biopsy or combined biochemical and imaging studies. All the participants were 18 years and older and those with known chronic kidney disease (estimated GFR < 60 ml/min), with untreated multifocal hepatocellular carcinomas, taking immunosuppressive drugs, or those who underwent liver transplantation were excluded. Because serum creatinine as well as liver function test every 3–4 months are included in the routine laboratory measurements in these patients, we enrolled only patients who showed stable renal function at least 3 months before admission. Patients who already showed ≥50% or 0.3 mg/dL of baseline serum creatinine at the time of admission were excluded (Fig. 1). Standard treatments including discontinuation of diuretics, volume resuscitation, therapeutic paracentesis with intravenous albumin infusion, antibiotics, high dose albumin with terlipressin in cases of suspected hepatorenal syndrome were administered according to patient needs and followed up until hospital discharge.

### Measurement

Upon admission, a urine sample was collected and stored at −70 °C after centrifuging for NGAL, TIMP2 and IGFBP7 measurements. Baseline demographic factors, clinical parameters including MELD and Charlson comorbidity index (CCI) scores, and plasma cystatin C were also determined. Routine blood and urine tests were serially performed every 2–3 days and AKI was diagnosed and staged according to the kidney disease improving global outcomes (KDIGO) criteria using only the serum creatinine, not urine output criterion. Etiologies of AKI was determined in the clinical context. History of volume contraction, bleeding, fever or exposure to nephrotoxicants were recorded. Findings from physical examination such as hypotension, tachycardia, tachypnea or laboratory findings including complete blood count, bacteriologic culture studies were also recorded. All these factors were considered to determine the possible etiologies of AKI. Baseline renal function was defined as a stable serum creatinine level within the last 3 months at the time of study enrollment.

### Statistical analysis

Results were expressed as mean ± SD when representing standard distribution or median with ranges for elements with skewed distribution. The SPSS software (IBM, version 22.0) was used for the statistical analyses. Comparisons between the 2 groups were performed using the t-test for numerical data, and the chi-square test for categorical data. Categorical variables were expressed as numerical proportions. Comparisons between multiple groups were analyzed using analysis of variance, followed by the Bonferroni post-hoc test. We conducted univariate and multivariate logistic regression analyses to assess the predictors of AKI and mortality. A conventional receiver operating characteristic (ROC) curve was generated for plasma cystatin C, urine [TIMP-2].[IGFBP7], and NGAL. We used MedCalc software version 17.7.2 DeLong method to compare the effectiveness of ROC between [TIMP-2]∙[IGFBP7] and NGAL. A p-value < 0.05 was considered statistically significant.
